# Correction to ‘Starvation effects on nitrogen and carbon stable isotopes of animals: an insight from meta-analysis of fasting experiments’

**DOI:** 10.1098/rsos.171551

**Published:** 2017-11-15

**Authors:** Hideyuki Doi, Fumikazu Akamatsu, Angélica L. González

*R. Soc. open sci.* **4**, 170633. (Published Online 23 August 2017). (doi:10.1098/rsos.170633)

Throughout the abstract, §§1–4 and the figures and tables, large delta characters (Δ) were incorrectly displayed as small delta characters (*δ*), instead of large italic delta characters (*Δ*). The correct text, figures and tables are presented below. The references are not included as they were presented correctly in the published paper.

Nitrogen and carbon stable isotopic compositions (*δ*^15^N and *δ*^13^C) of consumers have been used for physiological and food web studies. Previous studies have shown *δ*^15^N and *δ*^13^C values are affected by several biological and environmental factors during starvation, but the generality of the effect of starvation on *δ*^15^N and *δ*^13^C values has not yet been tested. Here, we performed a meta-analysis to evaluate the effects of starvation on *δ*^15^N and *δ*^13^C values of consumers, and the underlying factors that may explain the observed variation. The *Δ*^15^N and *Δ*^13^C values were calculated as the differences between the final *δ*^15^N and *δ*^13^C values of consumers (post-starvation) and the pre-starvation values on each experiment. Our meta-analysis showed a large variation in the *Δ*^15^N and *Δ*^13^C values of consumers (*Δ*^15^N range: –0.82 to 4.30‰; mean: 0.47‰ and *Δ*^13^C range: –1.92 to 2.62‰; mean: 0.01‰). The *Δ*^15^N values of most consumers increased along the length of the starvation period and were influenced by nitrogen excretion and thermoregulation types, probably because differences in nitrogen metabolism and thermoregulation affect nitrogen processing and excretion rates. None of our predictor variables accounted for the variation in *Δ*^13^C values, which showed both increases and decreases due to fasting. Our findings suggest that starvation results in changes in consumer *δ*^15^N values which are mainly explained by the length of the fasting period and by nitrogen and energy metabolism, but the underlying mechanisms of the starvation effects on *δ*^13^C values seem to be more complex than previously thought.

## 1. Introduction

Natural variation in nitrogen (^15^N/^14^N) and carbon (^13^C/^12^C) stable isotope ratios has provided important insights into food web structure and biogeochemical processing of nitrogen (N) and carbon (C) within ecosystems [1–4]. This is because the manner in which the stable isotopic composition of N (*δ*^15^N) and C (*δ*^13^C) of a consumer reflects its diet is fairly predictable [2,5,6]. These predictable differences in the *δ*^15^N and *δ*^13^C values between a consumer and its food resources have been called ‘trophic enrichment’ or ‘discrimination factor’ and their average values are useful to infer the trophic position of an animal and sources of energy [2]. Several meta-analyses and reviews [2,5,7,8], however, have reported a large variation in trophic enrichment across consumers. This isotope variation can impose difficulties when using average enrichment values to estimate trophic position, and hence there is still an increasing need to fully understand the underlying mechanisms affecting consumer isotope enrichment [9].

Many feeding experiments have been conducted to estimate the sources of variation in the *δ*^15^N and *δ*^13^C values of consumers and their prey [9]. These studies have shown that several important physiological, life-history and environmental factors can affect the *δ*^15^N and *δ*^13^C values of consumers and their trophic enrichment. For example, several studies have shown that the isotopic composition of a consumer changes during metamorphosis because of an increase in the excretion of ^15^N depleted meconium and frass [10,11]. Further, consumer nutritional status, such as N-poor resources or starvation, can cause an increase in *δ*^15^N and *δ*^13^C values of consumers [10].

Starvation is a state when animals do not eat, and therefore animal physiology and behaviour change [12]. During starvation, N and C uptake is near zero, but N and C loss by excretion and respiration remains, even if at a low rate [13–15]. Further, starvation can induce shifts in protein and carbohydrate metabolism [12], increasing the *δ*^15^N and *δ*^13^C values of consumers (see the papers in electronic supplementary material, table S1). These increases in *δ*^15^N values with starvation are likely due to an increased reliance on internal N resources with a large use and excretion of ^14^N [10,13,16]. Similarly, increases in animal *δ*^13^C values are caused by a strong reliance on internal lipid reserves during fasting; as lipids are depleted in ^13^C, the bulk *δ*^13^C values of consumers become heavily enriched [17,18]. Despite recent progress on the effects of starvation on consumer isotope composition, there is still little consensus about the general trends because studies have shown increases, decreases or lack of changes in both *δ*^15^N and *δ*^13^C values during fasting [9,19]. Reasons for these differences are unclear, but they may be dependent on the starvation-discrimination effects of the taxonomic group or the length of starvation period considered in these studies. Therefore, the generality of starvation effects on *δ*^15^N and *δ*^13^C values of consumers is currently unknown.

Here, we present the results of a meta-analysis testing the generality of starvation effects on the *δ*^15^N and *δ*^13^C values of consumers across aquatic and terrestrial systems. We hypothesized that the effect of starvation on the isotope composition of consumers would be predictable based on the following underlying mechanisms:

(1) Fasting time (experimental length used on the starvation experiment) influences the *δ*^15^N and *δ*^13^C values of consumers. Assuming that splanchnic organs represent up to 10% of all body protein [20], a 4% increase in whole organismal *δ*^15^N values will lead to an increase of only 0.4‰ in all body protein. Longer fasting time increases the use of body protein to obtain energy, with a preferential use of ^14^N, and thus the *δ*^15^N values of the remaining body protein increase. Based on this information, Martinez del Rio & Wolf [21] proposed a hypothesis that predicts that the *δ*^15^N values of consumers should increase with fasting time duration. This hypothesis has been supported by several empirical experiments [10,22]; however, to our knowledge, its generality has never been tested. In addition, animal body lipids depress bulk *δ*^13^C values because fatty acids have lower *δ*^13^C values than bulk *δ*^13^C values [23,24], and therefore, starvation may decrease the lipid reserves in the body of animals increasing ^13^C in consumers [25].
(2) Turnover rates of N and C are influenced by the metabolism of consumers. Metabolic theory of ecology (MTE) has provided the mechanistic basis to understand the fundamental role of metabolism in the ecology of organisms to ecosystems [26–28]. MTE can help predict the whole metabolic rate of an organism from its body mass and temperature [26]. Since metabolic rate relates to all biological processes [26], changes in metabolic rate would affect the isotope values of consumers, including isotopic incorporation and turnover rates potentially through the changes in the rates of excretion and use of body energy reserves. Increases in metabolic rate should be translated into decreases in *δ*^15^N and *δ*^13^C values of consumers.
(3) Other biological/ecological traits of organisms would affect the *δ*^15^N and *δ*^13^C values of consumers. For example, nitrogen excretion (e.g. type of nitrogenous waste or N waste), ontogenetic stage and thermoregulation could all affect isotope trophic enrichment. Types of nitrogenous waste include three chemical compounds: ammonia, urea and uric acid. The nature of N wastes as part of N metabolism affects the *δ*^15^N values of excretion [7], thus differences in types of nitrogenous waste of animals would affect bulk *δ*^15^N values during starvation. Ontogenetic stage can affect the *δ*^15^N and *δ*^13^C values of animals; juveniles allocate energy and materials to their growth beside maintenance and storage, and this has shown to increase *δ*^15^N values and decrease *δ*^13^C values of consumers [11,29]. Several lines of evidence have shown that tissue turnover rates in mammals, birds and fishes correlate with field metabolic rates, consequently affecting the *δ*^15^N and *δ*^13^C values of animals [30,31]. Thermoregulation affects metabolic processes [26], and can play a large role in the turnover of N and C in the tissue of endotherms and ectotherms, thus such differences in metabolism due to thermoregulation may influence the *δ*^15^N and *δ*^13^C values of animals during starvation.

## 2. Material and methods

### 2.1. Data sources

We performed a systematic and broad-range search for all publications in ISI Web of Science and Google Scholar using the search terms ‘isotope AND starv* OR fasting’, ‘isotope AND experiment*’, ‘isotope AND hung*’. The search initially returned 428 and 5580 hits from ISI Web of Science and Google Scholar, respectively. From these references, 123 papers were focused on the effects of starvation on stable isotopes, but we only kept those studies providing *δ*^15^N and/or *δ*^13^C values of consumers under starvation and control (pre-starvation) conditions in laboratory experiments (a total of 19 papers). To make sure that we included all available papers, we also reviewed the references from all relevant studies and reviews published on the starvation–stable isotopes effects to identify additional studies.

Data from the papers were collected from the texts, figures and tables of the papers. To gather data from figures, we used PlotDigitizer X v. 2.0.1 software (available: http://www.surf.nuqe.nagoya-u.ac.jp/~nakahara/Software/PlotDigitizerX/). In total, we obtained 47 starvation experiments from our total of 19 papers (electronic supplementary material, table S1). If several studies involved the same species, the result for each species was considered one estimate; therefore, some species were represented by multiple data points (see electronic supplementary material, table S1).

We obtained the experiment lengths (days), and initial and final body mass of the consumers from each study. We collected additional information of each species in the dataset (i.e. species traits), including taxonomic group (birds, fishes, mammals, reptiles and invertebrates), tissue type (whole body and some body parts as multiple samples), inhabiting ecosystem types (aquatic or terrestrial), nitrogen excretion (ammonia, urea or uric acid), ontogenetic stage (juvenile or adult) and species longevity. This additional information was collected from each paper or via Internet data sources. Few studies contained the isotopic values from the different body parts; but we only considered data from the whole body as the isotope data from the species. We obtained species mean body mass to calculate metabolic rate (see below) and thermoregulation types (ectotherm or endotherm) to evaluate the effects of metabolic rates and thermoregulation on the *δ*^15^N and *δ*^13^C values.

### 2.2. The *Δ*^15^N and *Δ*^13^C values of consumers and experiment length

The N and C isotopic discrimination (i.e. *Δ*^15^N or *Δ*^13^C values) of each consumer due to starvation was calculated as the differences between the final *δ*^15^N or *δ*^13^C values (post-starvation) and the initial *δ*^15^N or *δ*^13^C values (pre-starvation), respectively, as *ΔX *= **δ*X*_post-starvation_ − **δ*X*_pre-starvation_, where *X* is ^15^N or ^13^C. To account for the effects of experiment length (in days, from 5 to 243 days) on species lifespan, we calculated the standardized experiment length as (experiment length (day)/species longevity (day)).

### 2.3. Calculation of metabolic rate and magnitude of body mass loss

The metabolic rate (*B*, unit: Watt) can be predicted by the following equation [26]: *B* = *b*_0_*M*^0.75^e^–*E*/*kT*^, where *b*_0_ is the normalization constant independent of body size and temperature, and *M*, *E*, *k* and *T* are body mass (grams), the activation energy, Boltzmann's constant (8.62 × 10^−5^ eV K^−1^) and absolute body temperature in kelvin, respectively. Mean body temperature of endotherms was collected from the literature and online data sources. For ectotherms, ambient air or water temperatures on the laboratory setting were used to estimate the body temperature of the individuals. We estimated the magnitude of body mass loss by the difference between the final body mass after the experiment and the initial body mass before the experiment.

### 2.4. Statistical analyses

Differences in the *Δ*^15^N values among taxa group (birds, fish, invertebrates, mammals and reptiles) were independently tested by analysis of covariance (ANCOVA) using taxa group as a fixed factor, and experiment length, standardized experiment length, metabolic rate and mass loss as covariates.

To evaluate the effect of starvation on the *Δ*^15^N or *Δ*^13^C values of consumers, we performed generalized linear mixed models (GLMMs) [32], with a Gaussian distribution as the error distribution. In the models, we included metabolic rate, experiment length (days), standardized experiment length (days), magnitude of body mass loss from initial condition, inhabiting ecosystem types, nitrogen excretion, ontogenetic stages and thermoregulation types as fixed factors, and ‘species’ and ‘study’ were treated as random factors to account for variations among species and studies. For *Δ*^13^C values of consumers, we included lipid extraction (extracted or not) as a fixed effect for the GLMM. We ran a Shapiro–Wilk normality test on the effects of experiment length, standardized experiment length and metabolic rate after log_10_-transforming data. Before the GLMMs, we calculated the variance inflation factor (VIF) to check for colinearity of the factors. The maximum VIF was 2.37 for all models, indicating that colinearity among the factors would not significantly influence the results of GLMMs. For the GLMMs, we also performed the model selection of the explanatory variables through backward stepwise procedure using Akaike information criteria (AIC) and obtained the best model. All statistics and graphics were performed using R v. 3.3.1 [33], and for GLMMs, *t*-tests and graphics we used ‘lme4’, ‘lmerTest’ and ‘lattice’ packages for R, respectively. For all statistical tests, a value of 0.05 was used for determining statistical significance.

## 3. Results

### 3.1. Variations in *Δ*^15^N and *Δ*^13^C values of consumers

The *Δ*^15^N and *Δ*^13^C values of consumers across all experiments ranged from –0.82 to 4.30‰ and from –1.92 to 2.62‰, respectively (figure 1*a*,*b*). More than 100 of 139 *Δ*^15^N data of consumers (79%) were positive values, indicating that the *Δ*^15^N values of consumers increased along the starvation period in most of consumers. The range of variation of *Δ*^13^C values of consumers was larger than that of *Δ*^15^N values, and the *Δ*^13^C values distributed evenly from positive to negative values. The mean *Δ*^15^N and *Δ*^13^C values of consumers pooled across all starvation experiments were 0.47 ± 0.72‰ and 0.01 ± 0.78‰ (mean ± 1 s.d.), respectively.

**Figure 1.**
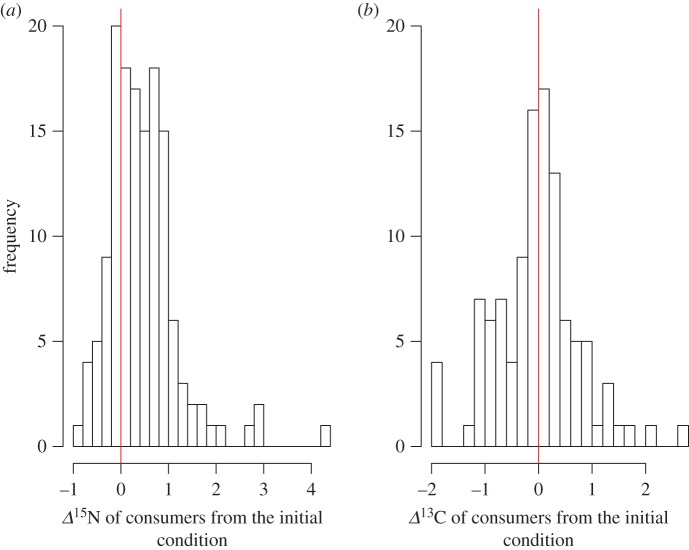
Frequency histograms of the values in the *Δ*^15^N (*a*) and *Δ*^13^C (*b*) values of consumers during starvation. The red line shows zero values of *Δ*^15^N and *Δ*^13^C.

### 3.2. Mechanisms underlying variation in *Δ*^15^N and *Δ*^13^C values of consumers

The *Δ*^15^N values of consumers showed significant starvation time-dependent changes in all taxonomic groups, with increases in *Δ*^15^N values with increasing experiment length and standardized experiment length (figure 2*a*,*b*, table 1). The slopes for the relationships between *Δ*^15^N values and experiment length or standardized experiment length were significant within taxa groups (ANCOVA, *F*_6,107 _= 5.11, *p* < 0.001 for experiment length, and *F*_6,107_ = 7.39, *p* < 0.001 for standardized experiment length), but not different among taxa groups (*F*_6,107 _= 0.795, *p* = 0.531 for experiment length, and *F*_6,107_ = 0.296, *p* = 0.880 for standardized experiment length). There were no significant effects of metabolic rate on the responses of *Δ*^15^N values to starvation for any of the taxonomic groups analysed (ANCOVA, *F*_1,63 _= 0.763, *p* = 0.386; figure 2*c*, table 1). The *Δ*^13^C values of consumers showed no significant starvation time-dependent changes (figure 3*a*,*b*). Similarly, we did not find any significant effect of metabolic rate on the effects of fasting on the *Δ*^13^C values of consumers (figure 3*c*, table 2). The magnitude of body mass loss had no significant effects on the responses of *Δ*^15^N and *Δ*^13^C values of consumers to starvation (figure 4*a*,*b*). Further, there were no significant differences in the slopes of *Δ*^15^N and *Δ*^13^C values and the magnitude of body mass loss (coefficients < –0.149, *p* = 0.611).

**Figure 2.**
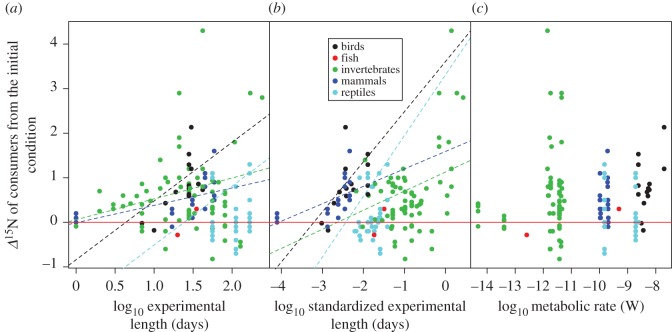
Relationships between the *Δ*^15^N values of consumers from the initial condition and the predictors including the experiment length (*a*), standardized experiment length (*b*) and metabolic rate (*c*). This last was calculated using the size of consumers following the MTE equation. The dashed lines show the significant regression lines from the GLMMs. The red line indicates zero values of *Δ*^15^N.

**Figure 3.**
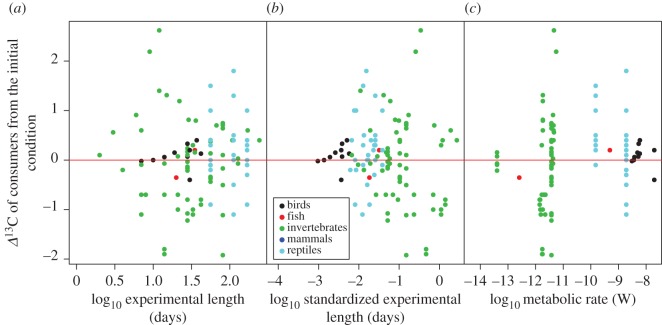
The relationships between the *Δ*^13^C values of consumers from the initial condition and the predictors including the experiment length (*a*), standardized experiment length (*b*) and metabolic rate (*c*). This last was calculated using the size of consumers following the MTE equation. There were no significant relationships between the *Δ*^13^C of consumers from the initial condition and the predictors in the GLMMs. The red line indicates zero values of *Δ*^13^C.

**Figure 4.**
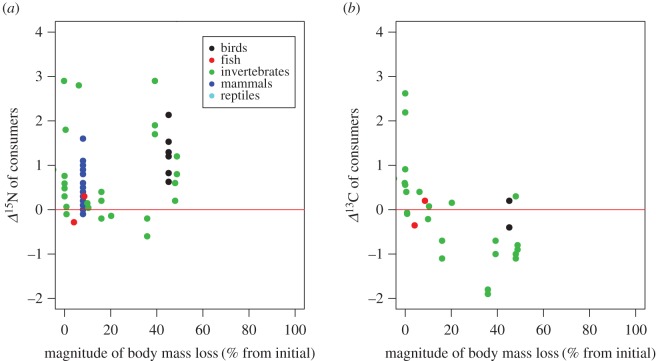
Relationships between the *Δ*^15^N (*a*) and *Δ*^13^C (*b*) values of consumers from the initial condition and mass loss rate at the end of experiments from the initial weights. The red line indicates zero values of *Δ*^15^N and *Δ*^13^C.

**Table 1.** Results of the full and best GLMMs for testing the effects of experiment length, standardized experiment length, consumer metabolic rate, magnitude of body mass loss, type of nitrogenous waste, thermoregulation type, ontogenetic stage and ecosystem type on *Δ*^15^N values of consumers. s.e. means standard error of the coefficient. The values in italics indicate significant factors (*p* < 0.05).

	full model					best model				
	coefficient	s.e.	*t*-value	*p*-value	AIC	coefficient	s.e.	*t*-value	*p*-value	AIC
experiment length	0.458	0.246	1.865	0.089	307.5	*0*.*482*	*0*.*231*	*2*.*083*	*0*.*042*	218.7
standardized experiment length	0.285	0.151	1.880	0.191		*0*.*462*	*0*.*201*	*2*.*295*	*0.041*	
metabolic rate	0.169	0.098	1.715	0.147		0.171	0.115	1.482	0.156	
magnitude of body mass loss	−0.149	0.274	−0.544	0.611						
type of nitrogenous waste	−0.877	0.466	−1.880	0.073		−0.887	0.466	−1.902	0.068	
thermoregulation	0.666	0.450	1.480	0.165		0.804	0.424	1.895	0.078	
ontogenetic stage	−0.365	0.453	−0.805	0.442						
ecosystem type	0.334	0.366	0.933	0.364						
(intercept)	0.668	0.475	1.407	0.224		1.226	0.618	1.984	0.069

**Table 2.** Results of the full and best GLMMs for testing the effects of experiment length, standardized experiment length, consumer metabolic rate, magnitude of body mass loss, type of nitrogenous waste, thermoregulation type, ontogenetic stage, ecosystem type and lipid extraction on the *Δ*^13^C values of consumers. s.e. means standard error of the coefficient. The best model, which was selected by AIC, was the full model.

	full/best model			
	coefficient	s.e.	*t*-value	*p*-value
experiment length	0.333	0.536	0.621	0.550
standardized experiment length	0.200	0.476	0.419	0.691
metabolic rate	0.095	0.266	0.356	0.735
magnitude of body mass loss	−0.137	0.506	−0.272	0.788
type of nitrogenous waste	−0.218	0.753	−0.289	0.776
thermoregulation	−0.370	0.988	−0.374	0.718
ontogenetic stage	0.305	1.124	0.272	0.790
ecosystem type	0.568	0.568	0.087	0.932
lipid extraction	−1.139	0.886	−1.285	0.145
(intercept)	0.030	1.308	0.023	0.983

The *Δ*^15^N values of consumers were marginally different between thermoregulation types (ectotherm or endotherm) of the consumers (figure 5*a*, table 1) and nitrogen excretion (ammonia, urea and uric acid; figure 5*b*); these factors were selected in the best model. We did not detect any significant effect of the ontogenetic stage and ecosystem type on the *Δ*^15^N values of consumers in the full and best models (electronic supplementary material, figure S1; table 1). Similarly, there were no significant differences in the *Δ*^13^C values of consumers grouped by type of nitrogenous waste, thermoregulation type, ontogenetic stage or ecosystem type (figure 5, table 2; electronic supplementary material, figures S1, S2), and none of these factors were selected in the best model (table 2). However, lipid treatment had the highest coefficient value in the model, and *Δ*^13^C values of consumers with non-lipid extraction were slightly higher than those with lipid extraction (table 2; electronic supplementary material, figure S2).

**Figure 5.**
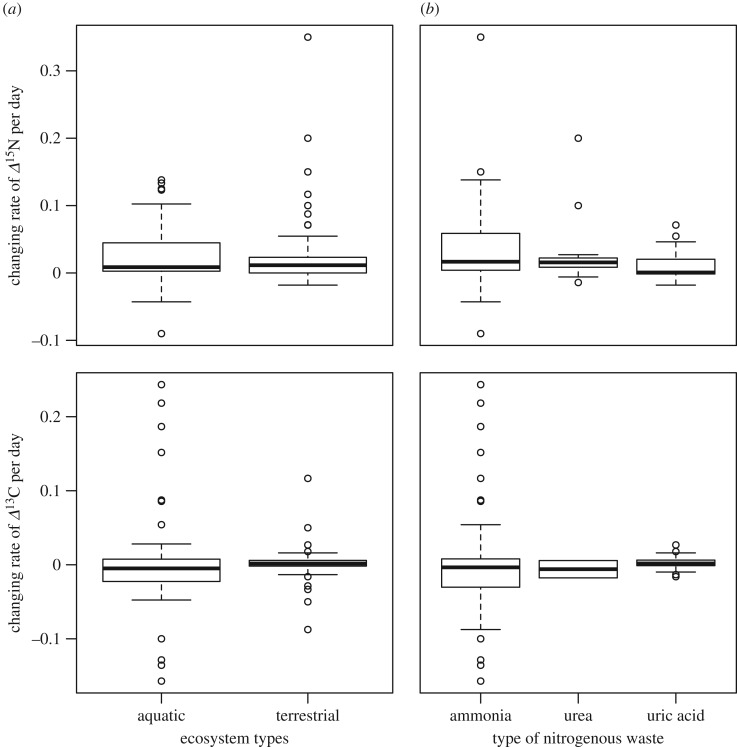
The *Δ*^15^N and *Δ*^13^C values of consumers related to thermoregulation (*a*) and type of nitrogenous waste (*b*) of the animals. Boxes in the box plot indicate median ± quartiles, and points indicate the outliers.

## 4. Discussion

Our meta-analysis showed large variations in the *Δ*^15^N and *Δ*^13^C values of consumers in relation to starvation. Overall, *Δ*^15^N values of most consumers increased, while *Δ*^13^C values showed both decreases and increases, along starvation length. Overall, this meta-analysis supports our predictions on the effect of fasting time duration (i.e. experiment length), nitrogen excretion and thermoregulation influencing the *Δ*^15^N values of consumers. However, contrary to our predictions, consumer *Δ*^15^N values were not affected by metabolic rate, mass loss, ecosystem type or ontogenetic stage. Similarly, none of our predictor variables accounted for the large variation in the *Δ*^13^C values of consumers.

### 4.1. Mechanisms underlying variation in *Δ*^15^N and *Δ*^13^C values of consumers

We found that fasting time affected the *Δ*^15^N values by increasing *δ*^15^N values of consumers. During starvation, there is a strong dependency on an internal recycling of N, and the N reserves in the body (i.e. protein) are used to get energy [22]. During this process, ^14^N is primarily used and excreted [22]. Thus, the bulk *δ*^15^N values in the body of consumers become enriched in ^15^N [10,22]. Also, fasting animals conserve protein when they fast, especially if they have adequate reserves [34–37]. Such differences in conversion rate of protein to energy may induce the variation in the *Δ*^15^N values of consumers. Our results are consistent with the hypothesis proposed by Martinez del Rio & Wolf [21], who theoretically predicted that the *δ*^15^N values of consumers should increase with the length of fasting.

The lack of significant effects of metabolism and magnitude of body mass loss on the *Δ*^15^N values of consumers may indicate that the turnover rate of the N isotopes is not an important factor during starvation (but see MacAvoy *et al*. [30]). This is probably because the effect of turnover rates on N isotopic discrimination was relatively smaller than that of the starvation length. In addition, several lines of evidence suggest that although the relationship between metabolic rate and isotope discrimination is mediated through protein metabolism, a decoupling between these processes is possible because protein catabolism is not the only source of energy during fasting [38].

Nitrogen excretion and thermoregulation were important factors affecting the *Δ*^15^N values of consumers during starvation. The role of N excretion on isotopic discrimination may be due to the differences in N metabolism [7]. Different N waste products go through a series of steps from ammonia to urea or uric acid; these steps likely fractionate ^14^N and ^15^N at different rates [7]. According to Vanderklift & Ponsard [7], animals excreting urea and uric acid would show larger consumer-diet ^15^N enrichment than those excreting ammonia. Our results showed that ammonia-excreting animals have enriched ^15^N (compared with urea and uric acid) under starvation conditions. The additional series of steps after ammonia formation for the conversion and excretion of nitrogenous wastes as urea and uric acid likely explain this pattern.

We also found that thermoregulation type was a major factor affecting *Δ*^15^N values of consumers during starvation. Food limitation typically slows down metabolic rates and affects the costs of thermoregulation. During starvation, the thermoregulation costs, which are higher in endotherms than ectotherms, could deplete energy stores and may affect *δ*^15^N values due to changes in protein metabolism affecting isotope incorporation [27,39,40].

None of the hypothesized factors, including experiment length (5–243 days), significantly affected *Δ*^13^C values of consumers; however, lipid extraction had the strongest effect on the *Δ*^13^C values of consumers. Lipids in animals have lower *δ*^13^C values than bulk *δ*^13^C values and individual fatty acids have different *δ*^13^C values [23,24,41]. Multiple studies have shown that starvation results in an enrichment in ^13^C due to an increase in the amount of carbon from ^13^C-depleted lipids metabolized to meet energetic demands [42,43], others have shown no effects of fasting on *Δ*^13^C [44,45]. Our study revealed that the average bulk *δ*^13^C values treated with lipid extraction decreased after starvation rather than increasing, and thus, it may not simply result from removal of the lipids with relatively low *δ*^13^C values. These results are consistent with the later studies; however, most of the studies showing no effect of starvation over isotopic composition evaluated bulk responses in *δ*^13^C values. For example, McCue *et al*. [46] showed that cockroach did not change significantly in response to starvation lasting up to 168 days, although the isotopic values of the excreta became significantly depleted in ^13^C. Similarly, Gaye-Siessegger *et al*. [17] found enriched *δ*^13^C values in the lipid-free material of fishes after starvation. Different *Δ*^13^C values of consumers in response to starvation due to lipid extraction treatments may be driven by differences in species compositions between extracted and non-extracted treatments, suggesting species-specific variations in lipid content. However, further studies are needed to test the hypothesis of the different *Δ*^13^C values of consumers treated for lipid extraction and no lipid extraction.

In this meta-analysis, we tested several factors including environmental conditions and biological traits that could explain variation in stable isotope composition of consumers under starvation. Nevertheless, some other factors not tested in this study, such as water and nutrient stresses, could also contribute to the variation of the starvation effects over stable isotopes [44,47]. During starvation experiments testing for isotope changes, the animals may have been exposed to water, nutrient and other stresses imposed by the experimental conditions [47]. However, the effects of such experimental conditions on the *δ*^15^N and *δ*^13^C values of animals were not tested in these experiments. We propose that further experiments need to account for the effect of these stressors on the isotope values of consumers during the starvation experiments.

### 4.2. Concluding remarks

Although our study showed variations in *Δ*^15^N and *Δ*^13^C values of consumers, which resulted from several of our predictors, including experiment length and consumer traits, we did not find straightforward mechanisms explaining the large variations in the *Δ*^13^C values of consumers. This lack of conclusive results may be due to the complex mechanisms underlying the starvation effects on the isotope values. In addition, the sample sizes in our meta-analysis were also limited, especially for invertebrates (nine taxa), which could have precluded us from finding conclusive trends. Thus, we encourage more studies to test the starvation-effect hypothesis, especially for invertebrate species.

